# Comparison of in vivo hindfoot joints motion changes during stance phase between non-flatfoot and stage II adult acquired flatfoot

**DOI:** 10.1186/s13047-022-00577-w

**Published:** 2022-10-13

**Authors:** Zhenhan Deng, Zijun Cai, Siyu Chen, Yan Liu, Fanglin Chen, Zhiqin Deng, Yusheng Li, Jian Xu

**Affiliations:** 1 Department of Sports Medicine, The First Affiliated Hospital of Shenzhen University, Shenzhen Second People’s Hospital, Shenzhen, Guangdong China; 2 Department of Orthopaedics, Xiangya Hospital, Central South University, Changsha, Hunan China; 3 Department of Critical Care Medicine and Infection Prevention and Control, The First Affiliated Hospital of Shenzhen University, Shenzhen Second People’s Hospital, Shenzhen, 518035 Guangdong China; 4 Department of Orthopedics, The Fourth Affiliated Hospital of Guangxi Medical University, Liuzhou Worker’s Hospital, Liuzhou, 545000 Guangxi China; 5 Hand and Foot Surgery Department, The First Affiliated Hospital of Shenzhen University, Shenzhen Second People’s Hospital, Shenzhen, Guangdong China; 6 Department of Orthopedics, The First Affiliated Hospital, Zhejiang University School of Medicine, Hangzhou, 310003 Zhejiang China

**Keywords:** Adult acquired flatfoot deformity, Movement, In vivo, Hindfoot joint

## Abstract

**Background:**

To compare the kinematic characteristics of hindfoot joints in stage II adult acquired flatfoot deformity (AAFD) with those of non-flatfoot through the 3D-to-2D registration technology and single fluoroscopic imaging system.

**Methods:**

Eight volunteers with stage II AAFD and seven volunteers without stage II AAFD were recruited and CT scans were performed bilateral for both groups in neutral positions. Their lateral dynamic X-ray data during the stance phase, including 14 non-flatfeet and 10 flatfeet, was collected. A computer-aided simulated light source for 3D CT model was applied to obtain the virtual images, which were matched with the dynamic X-ray images to register in the “Fluo” software, so that the spatial changes during the stance phase could be calculated.

**Results:**

During the early-stance phase, the calcaneous was more dorsiflexed, everted, and externally-rotated relative to the talus in flatfoot compared with that in non-flatfoot (*p* < 0.05). During the mid-stance phase, the calcaneous was more dorsiflexed and everted relative to the talus in flatfoot compared with that in non-flatfoot (*p* < 0.05); however, the rotation did not differ significantly between the two groups (*p* > 0.05). During the late-stance phase, the calcaneous was more plantarflexed, but less inverted and internally-rotated, relative to the talus in flatfoot compared with that in non-flatfoot (*p* < 0.05). During the early- and mid-stance phase, the navicular was more dorsiflexed, everted, and externally-rotated relative to the talus in flatfoot compared with that in non-flatfoot (*p* < 0.05). During the late-stance phase, the navicular was more plantarflexed, but less inverted and internally-rotated, relative to the talus in flatfoot compared with that in non-flatfoot (*p* < 0.05). There was no difference in the motion of cuboid between the two groups during the whole stance phase (*p* > 0.05).

**Conclusions:**

During the early- and mid-stance phase, excessive motion was observed in the subtalar and talonavicular joints in stage II AAFD. During the late-stance phase, the motion of subtalar and talonavicular joints appeared to be in the dysfunction state. The current study helps better understanding the biomechanics of the hindfoot during non-flatfoot and flatfoot condition which is critical to the intervention to the AAFD using conservative treatment such as insole or surgical treatment for joint hypermotion.

## Background

Adult acquired flatfoot deformity (AAFD) is a common degenerative disease with multiple stages that causes poor alignment of joints, which will lead to the generation of pain and ultimately affect the normal function and activity of the foot and ankle [1]. AAFD is a common progressive pathology that mainly affects patients after their 50 s [2]. Posterior tibial tendon dysfunction (PTTD) has long been recognized as a key causative factor even though its etiology has not been fully elucidated to date, while the great majority of patients have their talo-navicular joint (TNJ) subdislocated in one or more planes [2–4].

In 1989, based on the study of tibialis posterior tendon dysfunction, Johnson and Strom set a three-stage classification for AAFD [5]. Among them, Stage II AAFD refers to the transitional period from a flexible deformity to a stiff deformity, which is most widely studied in clinical practice but is also the most controversial one for its treatment method selection [1, 6, 7]. During this period, the chief clinical manifestations are early forefoot abduction deformity, midfoot collapse, and hindfoot valgus deformity. In the early stages of AAFD (Stages I, IIa and IIb), many treatment options focus on rebalancing the foot structure. However, when the deformity is rigid (stage III or IV), more restrictive treatments are preferred, such as arthrodesis of the hindfoot joints [8–10]. The anatomic abnormalities of hindfoot joints are closely related to the AAFD onset, while the disruption of the linkage mechanism is a major factor in its progression. Thus, the research on the characteristics of the motion of hindfoot joints could be of great importance. The hindfoot joints are mainly comprised of three joints, namely the subtalar joint (STJ), the TNJ and the calcaneo-cuboid joint (CCJ). Wang et al. detected synchronous and homodromous rotational motions of the TNJ, STJ, and CCJ during the stance phase [11]. Van de Velde et al. showed significant differences in range of motion in patients with flatfoot. They demonstrated that decreased mobility occurred mainly in the hindfoot and midfoot [12]. Hyuck Soo Shin et al. Showed flatfoot deformity affected the kinematics of the foot and ankle in proportion to the severity of deformity [13]. Few studies have compared hindfoot joints motion between non-flatfoot volunteers and stage II AAFD patients. Zhang et al. [14] reported excessive motion in the TNJ and STJ among AAFD patients, but the dynamic condition in the gait stance phase remained unclear. Wang et al. [11] found that TNJ had a greater mobility in the sagittal plane while STJ had a greater mobility in the coronal plane in stage II AAFD patients by applying the 3D-to-2D registration technique based on dynamic x-rays. Although this study analyzed the motion difference in the tarsal complex between non-flatfoot and flatfoot in the stance phase, it did not reflect the detailed motion change and trend during the whole stance phase, which can be divided into heel-strike (HS), foot-flat (FF), midstance (MS), heel-off (HO), and toe-off (TO) [15].

Our study, by utilizing the advanced three dimensional (3D)-to-two dimensional (2D) registration technique as well [11, 16–19], aimed to verify the accuracy and repeatability of this technique, investigate the motion characteristics of hindfoot joints in the stance phase more comprehensively based on the above mentioned 5 events in stage II AAFD. Understanding the biomechanics of the hindfoot is critical to the proper care of patients with a variety of orthopedic impairments and foot deformities resulting from conditions such as cerebral palsy, spina bififida, club foot, traumatic brain injury and spinal cord injury [20, 21].

## Methods

Seven adult volunteers with no clinical signs of flatfoot (4 males and 3 females; mean age: 44.3 ± 7.3 (range, 26 to 52) years) and 8 adult volunteers with stage II AAFD (4 males and 4 females; mean age: 41.2 ± 8.5 (range, 21 to 56) years) who were free of foot and ankle deformities, tumors, acute and chronic injuries, and gait abnormalities were enrolled in the present study. A total of 10 flatfeet were obtained and confirmed by CT scan in flatfoot group, including 4 left and 6 right feet, while the non-flatfoot group consisted of 14 feet. The sample size in the groups was calculated which can guarantee that we had enough power for the statistics analysis. All the recruited subjects had signed the informed consent form approved by the Institutional Review Board (IRB) of Liuzhou Worker’s Hospital prior to the formal research. The FluoMotion software (Innomotion Inc., Shanghai) was used to perform the 3D-to-2D registration.

The accuracy of the kinematic data measured by 3D model registration was validated on a cadaveric specimen obtained from a selected lower leg (male, 68 years old) by adopting the radiographic stereophotogrammetry analysis (RSA) technique.

### Accuracy and repeatability assessment of the 3D model and 2D image registration technique applied to the hindfoot by RSA

The RSA technique, with an accuracy ranging from 0.05 to 0.5 mm and a 0.15° turning range [18], was utilized to verify the accuracy as well as the reproducibility of the 3D-to-2D registration technique. This technique requires the implantation of a certain amount of tantalum beads at a fixed position inside the bone tissue or around an artificial joint prosthesis as a marker.

**Fig. 1:**
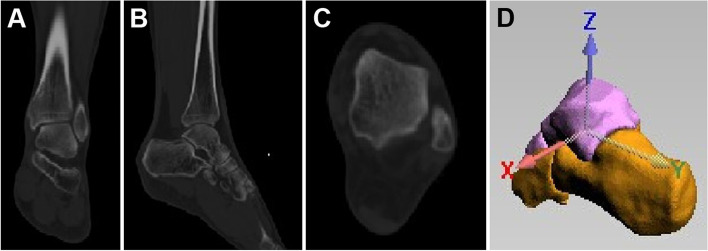
CT image of the ankle joint in coronal (**A**), sagittal (**B**) and transverse (**C**) views. (D) Setting of talus coordinate axis

Fresh intact calf specimens were obtained from a 68-year-old man and 4 tantalum beads with the size of 1 mm were implanted into the talus, calcaneus, navicular bone, and cuboid bone respectively for CT imaging. The scanning range was from the sole of the foot to 10 cm above the ankle, and the layer thickness was set as 0.67 mm. The images were imported into Mimics 17.0 software with coronal, sagittal and cross-sectional planes (Fig. 1A-C). Then, the 3D models were reconstructed. The virtual coordinates were constructed with the ankle in the neutral position (0 degree dorsiflexion) as a reference by regarding the center of mass of each bone as the origin of the coordinate system (Fig. 1D). The rotation in the coordinate system was considered as the dorsiflexion and plantarflexion around the X-axis, the inversion and eversion around the Y-axis, and the internal rotation and external rotation around the Z-axis respectively. The dorsiflexion, inversion, and internal rotation were defined as positive, whereas the plantarflexion, eversion, and external rotation were defined as negative.

**Fig. 2:**
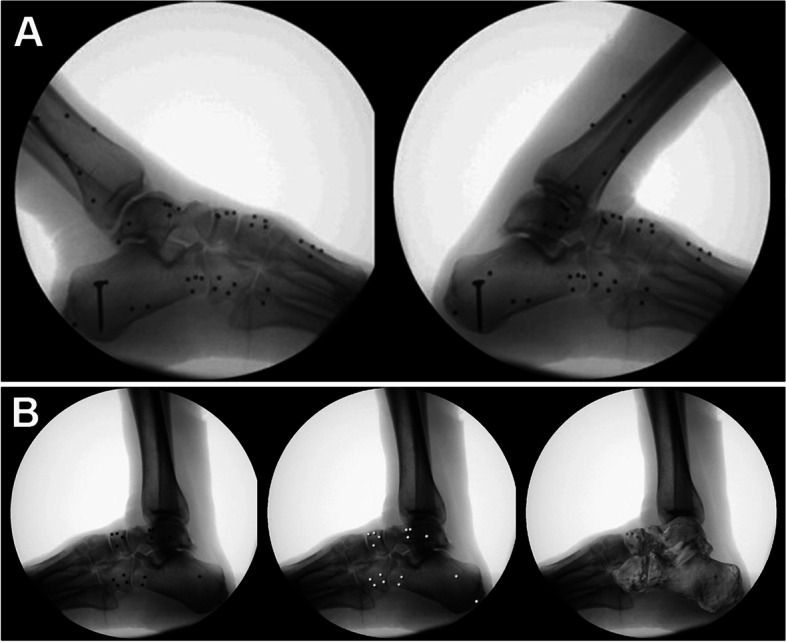
**A** X-ray later imaging of the ankle joint from plantar flexion to dorsiflexion of the specimen with tantalum beads implanted. **B** Matching of tantalum beads by software

The single plane X-ray imaging system consists of an X-ray emission device as well as an adjustable gait platform that can adjust the height to accommodate the height of the fluoroscopic C-arm machine. After fixing a calibration device in the receiver section of the C-arm machine, the foot of the specimen was placed onto a gait platform to artificially simulate its dynamic processes without load and to perform the lateral imaging of X-ray in real-time (Fig. 2A). The dynamic images were taken at a frequency of 10 Hz with 1000 × 1000 pixels for each image.

Five images were randomly selected for registration to verify the accuracy in the dynamic simulation process. The corresponding 3D model was matched to the respective bone block images on the radiographs for contouring (Fig. 2B). This software is able to automatically calculate the relative 3D translation and rotation of the bone blocks in the six degrees of freedom. Δ X, Δ Y, Δ Z represent the difference of translation on X, Y and Z axes respectively, and Δα, Δβ, Δγ represent the difference of rotation angle on X, Y and Z axes respectively.

### Dynamic measurements of hindfoot joints during the stance phase in non-flatfoot and stage II AAFD volunteers

**Fig. 3:**
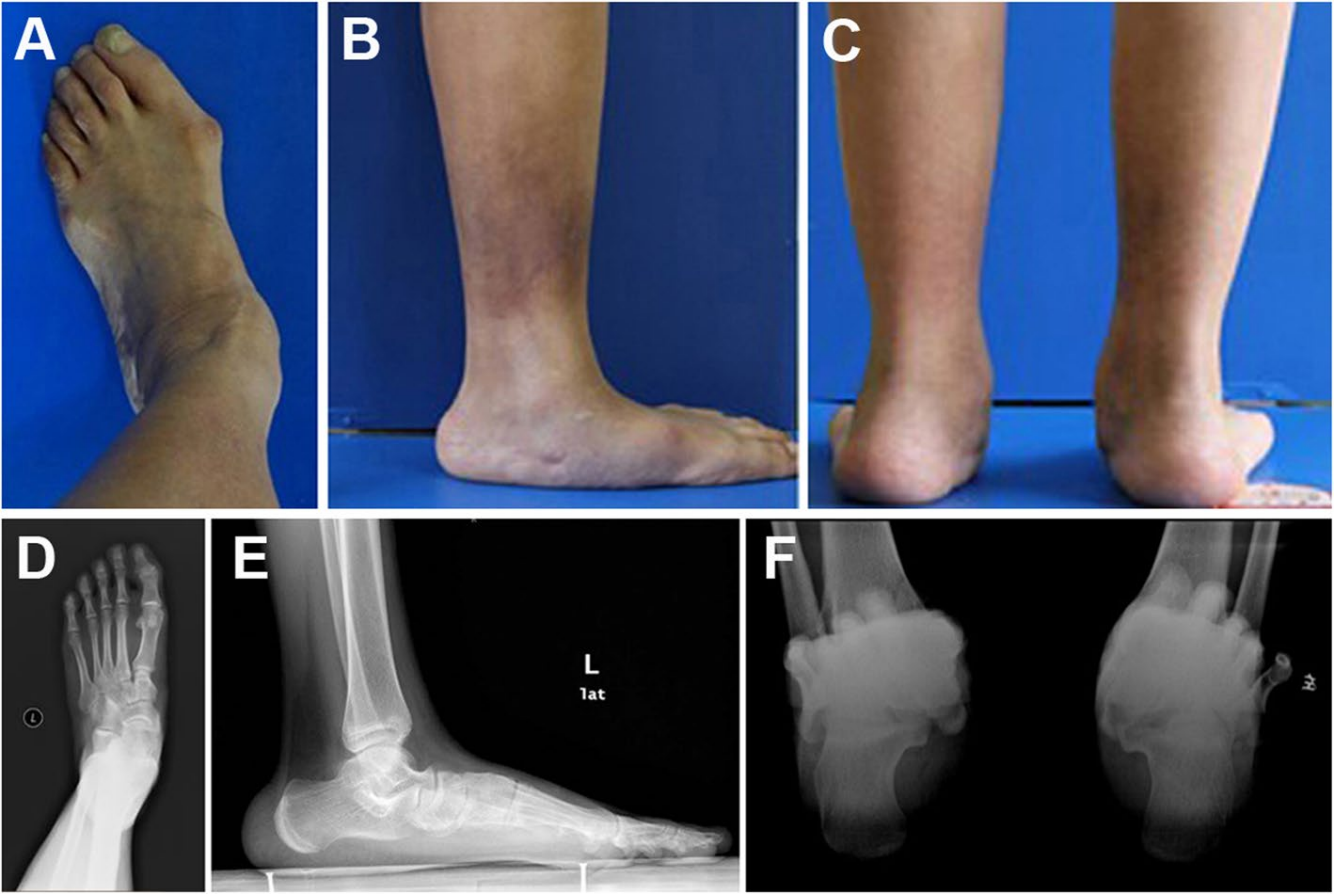
Macro view and X-ray of Stage II AAFD. **A**, **D** Forefoot abduction deformity. **B**, **E** Medial arch collapse. (**C**, **F**) Hindfoot valgus

**Fig. 4:**
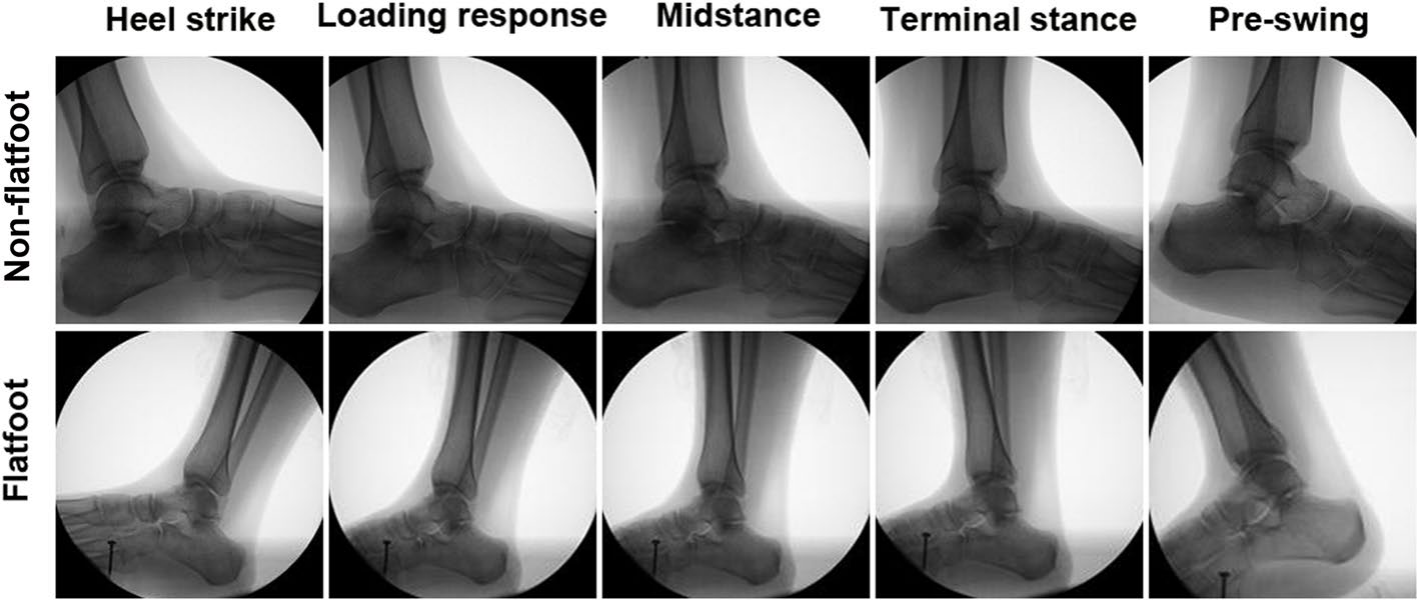
Key gait postures in the non-flatfoot and stage II AAFD

**Fig. 5:**
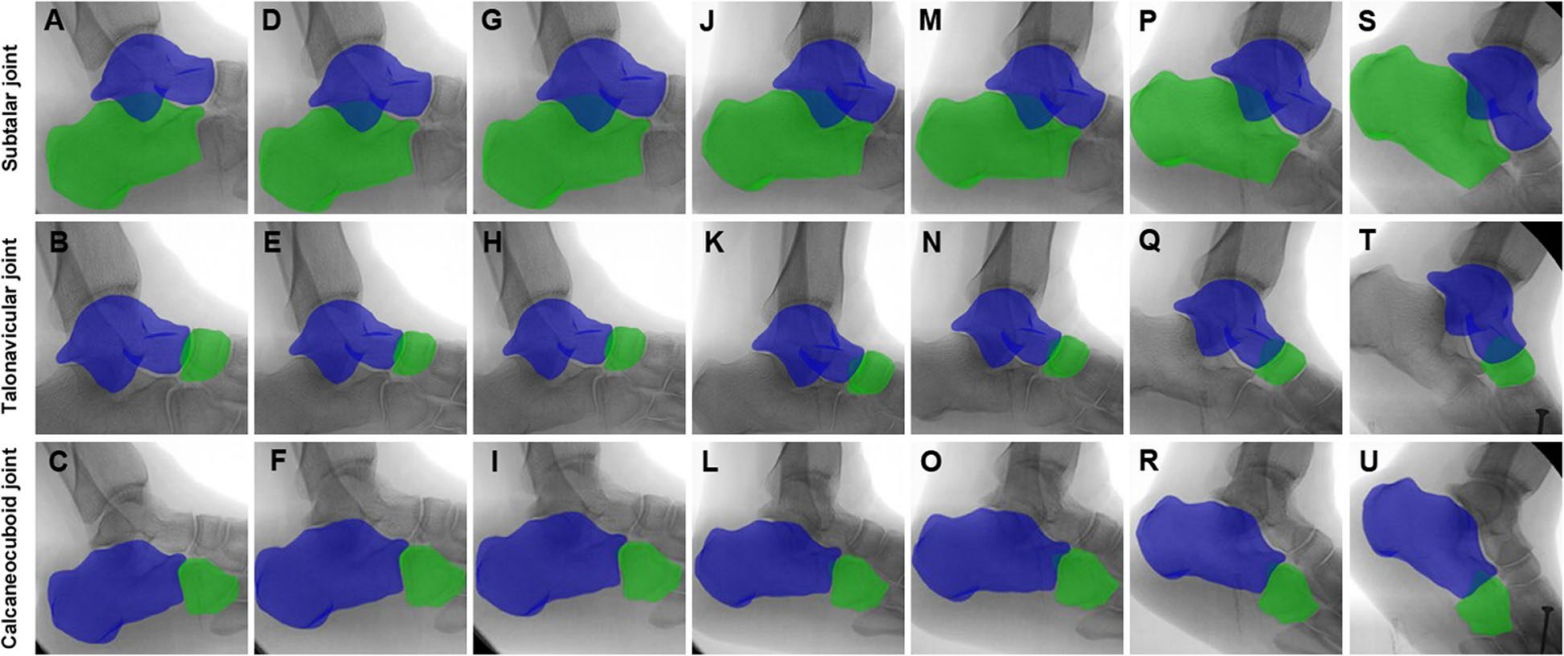
3D-to-2D matching of subtalar joint (A-S), talonavicular joint (B-T) and calcaneocuboid joint (C-U) in the 7 postures

CT images were collected for all participants from both groups with forefoot abduction deformity, medial arch collapse and hindfoot valgus (Fig. 3). All the volunteers were trained to walk for three consecutive steps in order to match the X-ray dynamic imaging capture system. The X-ray dynamic fluoroscopy was performed during the second step of walking. Before that, the gait speed should maintain at 0.5 m/s. Then, 7 key gait postures which consisted of the stance phase in all fluoroscopic images were picked up for registration analysis to compare between the flatfoot and non-flatfoot (Fig. 4). The motion (Fig. 5) in the hindfoot joints was calculated automatically by the software.

### Statistical analysis

All statistical data was analyzed by SPSS 20.0. The results were expressed by mean ± standard deviation. The motion of the foot segments were analysed by non-parametric tests (Mann–Whitney U test). The multivariate analysis of variance was used to perform pairwise comparison of the hindfoot joints in the stance phase between the non-flatfoot and flatfoot. When *p* < 0.05, a statistically significant difference was indicated.

## Results

### Verification of accuracy and repeatability of the registration of 3D model with 2D image in the single plane perspective system

The difference in the motion data of the bone block and hindfoot joints was calculated using the RSA technique and the 3D-to-2D image single plane registration technology (Tables 1 and  2). The talus had the largest rotation deviation, which can be up to -0.45 ± 0.7°. In any other planes perpendicular to this plane, the navicular had the largest translation deviation, which can be up to 3.07 ± 2.52 mm; the talus had the largest rotation deviation, which can be up to -0.87 ± 0.32°. In the hindfoot, the maximum translation deviation of the talonavicular joint in the same plane of X-ray imaging can be up to -0.13 ± 0.4 mm, while the talus had the largest rotation deviation, which can be up to -1.87 ± 1.05°. In any other planes perpendicular to this plane, the subtalar joint had the largest translation and rotation deviation, which can be up to 2.05 ± 0.95 mm and -1.8 ± 2.33°, respectively.

**Table 1:** Differences in hindfoot bone motion data measured by RSA technique and Fluo software single-plane registration technique

**Bone**	**Translation(mm)**						**Rotation (°)**					
	**∆x**	**P**	**∆y**	**P**	**∆z**	**P**	**∆α**	**P**	**∆β**	**P**	**∆γ**	**P**
**Talus**	3.00 ± 2.21	0.23	0.57 ± 1.73	0.29	-0.21 ± 0.38	0.47	-0.45 ± 0.7	0.65	0.48 ± 2.92	0.27	-0.87 ± 2.32	0.44
**Calcaneus**	1.5 ± 0.51	0.55	0.22 ± 0.4	0.67	-0.16 ± 0.41	0.91	-0.20 ± 0.23	0.34	0.86 ± 1.82	0.75	-0.27 ± 1.12	0.57
**Navicular**	3.07 ± 2.52	0.79	0.38 ± 1.12	0.46	-0.87 ± 0.90	0.48	-0.25 ± 1.25	0.37	0.90 ± 2.30	0.72	-0.70 ± 1.94	0.95
**Cuboid**	2.19 ± 2.4	0.77	0.63 ± 1.47	0.35	-1.57 ± 3.07	0.37	0.02 ± 0.9	0.48	0.16 ± 3.4	0.53	-0.70 ± 1.94	0.48

^*^P < 0.05 indicated a statistical difference

**Table 2:** Differences of hindfoot motion data measured by RSA technique and Fluo software single plane registration technique

**Joint**	**Translation(mm)**						**Rotation (°)**					
	**∆x**	**P**	**∆y**	**P**	**∆z**	**P**	**∆α**	**P**	**∆β**	**P**	**∆γ**	**P**
**Subtalar joint**	2.05 ± 0.95	0.51	-0.28 ± 0.19	0.42	0.19 ± 0.51	0.23	-1.87 ± 1.05	0.63	0.76 ± 2.24	0.19	-1.8 ± 2.33	0.61
**Talonavicular joint**	-1.01 ± 0.2	0.59	0.33 ± 0.4	0.67	0.27 ± 0.78	0.48	-0.24 ± 2.03	0.46	-1.0 ± 3.26	0.43	-0.17 ± 2.17	0.75
**Calcaneocuboid joint**	1.00 ± 0.72	0.37	-0.29 ± 0.38	0.56	-0.29 ± 0.86	0.60	0.56 ± 2.89	0.52	-0.12 ± 2.91	0.57	0.61 ± 2.69	0.83

^*^P < 0.05 indicated a statistical difference

### Average trend of the hindfoot joints motion

**Fig. 6:**
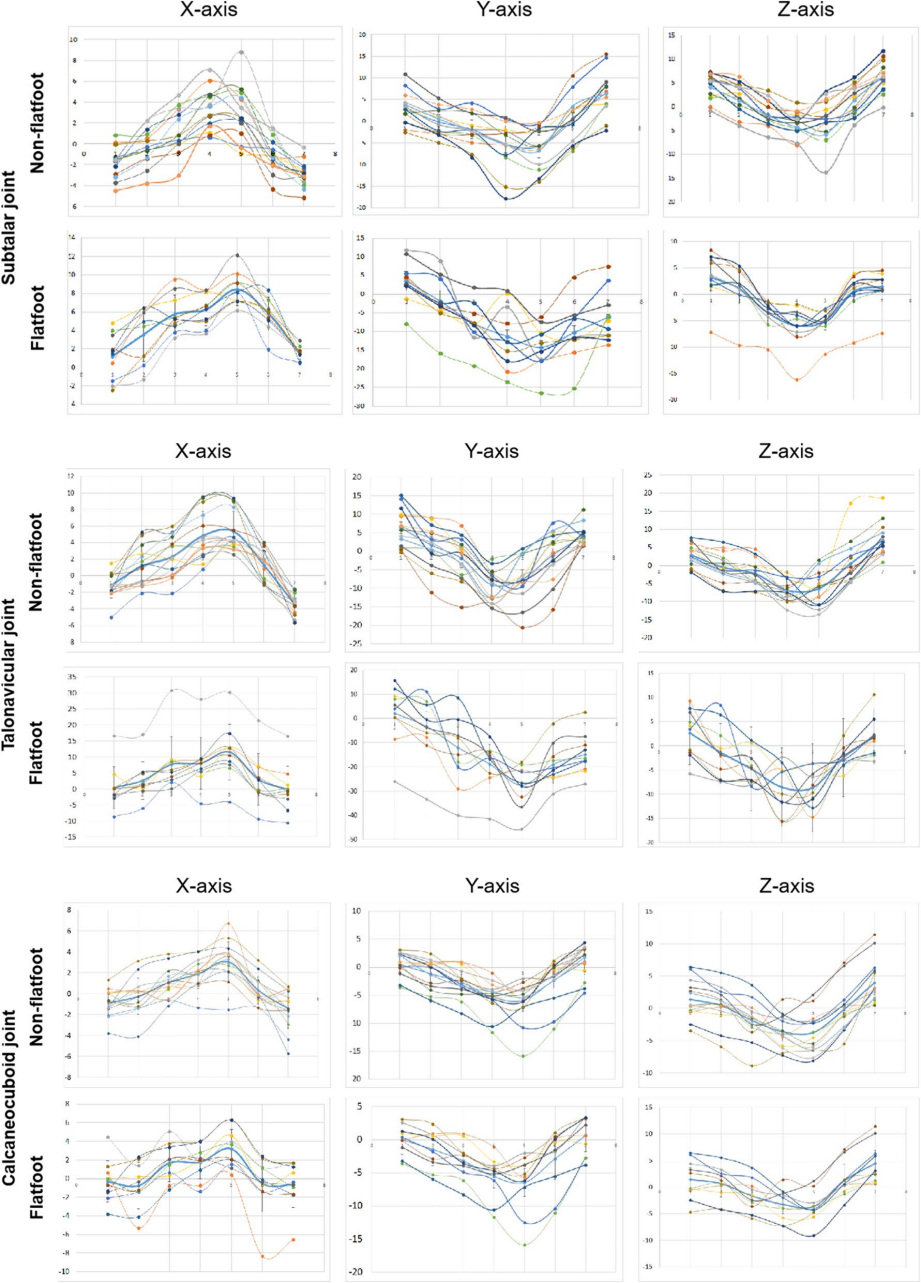
Rotation trend of subtalar joint, talonavicular joint, and calcaneocuboid joint of all non-flatfoot and flatfoot volunteers from the first posture to the seventh posture around X-, Y- and Z-axes

Figure 6 shows the trend of 7 key gait postures of each joint in the stance phase on the X, Y, and Z axes. Table 3 presents the comparison of the ROM of hindfoot between the non-flatfoot and stage II flatfoot. It can be seen that the flatfoot had a larger ROM in the subtalar joint on X-axis (8.11 ± 1.39° vs 6.93 ± 2.47°, *p* < 0.05) and Y-axis (19.77 ± 5.08° vs 13.65 ± 3.64°, *p* < 0.05) as well as a larger ROM in the talonavicular joint on X-axis (13.56 ± 4.14° vs 9.19 ± 2.86°, *p* < 0.05) and Y-axis (31.91 ± 8.45° vs 18.41 ± 3.80°, *p* < 0.05) than the non-flatfoot.

**Table 3:** Comparison of range of motion of hindfoot between the non-flatfoot and stage II flatfoot

	**ROM of subtalar joint (°)**			**ROM of talonavicular joint (°)**			**ROM of calcaneocuboid joint (°)**		
	X-axis	Y-axis	Z-axis	X-axis	Y-axis	Z-axis	X-axis	Y-axis	Z-axis
**Non-flatfoot**	6.93 ± 2.47	13.65 ± 3.64	10.74 ± 2.52	9.19 ± 2.86	18.41 ± 3.80	16.11 ± 4.44	4.89 ± 1.44	8.22 ± 2.3	9.11 ± 3.30
**Flatfoot**	8.11 ± 1.39	19.77 ± 5.08	10.12 ± 2.99	13.56 ± 4.14	31.91 ± 8.45	17.13 ± 5.38	5.29 ± 1.82	8.49 ± 2.68	9.45 ± 3.44
**P**	0.023*	< 0.001*	0.21	< 0.001*	< 0.001*	0.16	0.45	0.33	0.76

*ROM * Range of motion

*P* < 0.05 indicated a statistical difference

^*^P < 0.05 indicated a statistical difference

### ROM of hindfoot from the first to the third posture (HS to FF)

**Fig. 7:**
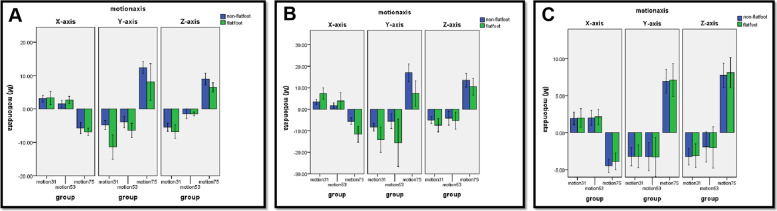
ROM changes of ankle joints of non-flatfoot and stage II AAFD (motiondata represents motion data; motionaxis represents motion axis; motion31, motion53 and motion75 represent the first to the third posture, the third to the fifth posture, and the fifth to the seventh posture respectively). **A** Subtalar joint. **B** Talonavicular joint. **C** Calcaneocuboid joint

Table 4 details the ROM of hindfoot from the first to the third posture (HS to FF). In the subtalar joint, the flatfoot had a higher ROM on all the X-axis (3.33 ± 2.78° vs 3.09 ± 1.72°, *p* < 0.05), Y-axis (11.40 ± 5.11° vs 4.80 ± 2.28°, *p* < 0.05) and Z-axis (6.76 ± 2.82° vs 5.45 ± 2.13°, *p* < 0.05) than the non-flatfoot (Fig. 7A). Similarly, in the talonavicular joint, the flatfoot also had a higher ROM on all the X-axis (7.27 ± 3.78° vs 3.45 ± 1.93°, *p* < 0.05), Y-axis (14.15 ± 8.20° vs 8.32 ± 3.30°, *p* < 0.05) and Z-axis (7.40 ± 4.36° vs 5.12 ± 2.86°, *p* < 0.05) than the non-flatfoot (Fig. 7B). However, in the calcaneocuboid joint, there was no difference between the non-flatfoot and flatfoot on X-axis (1.93 ± 1.49° vs 1.98 ± 1.77°, *p* > 0.05), Y-axis (3.22 ± 2.18° vs 3.20 ± 2.17°, *p* > 0.05) and Z-axis (3.24 ± 1.95° vs3.10 ± 2.21°, *p* > 0.05) (Fig. 7C).

**Table 4:** ROM of the hindfoot from the first to the third posture, from the third to the fifth posture, and from the fifth to the seventh posture

	**ROM of subtalar joint (°)**			**ROM of talonavicular joint (°)**			**ROM of calcaneocuboid joint (°)**		
	**Non-flatfoot**	**Flatfoot**	**P**	**Non-flatfoot**	**Flatfoot**	**P**	**Non-flatfoot**	**Flatfoot**	**P**
**Motion31(X)**	3.09 ± 1.72	3.33 ± 2.78	0.031*	3.45 ± 1.93	7.27 ± 3.78	0.001	1.93 ± 1.49	1.98 ± 1.77	0.57
**Motion31(Y)**	-4.80 ± 2.28	-11.4 ± 5.11	< 0.001*	-8.32 ± 3.30	-14.15 ± 8.20	0.001	-3.22 ± 2.18	-3.20 ± 2.17	0.65
**Motion31(Z)**	-5.45 ± 2.13	-6.76 ± 2.82	0.021*	-5.12 ± 2.86	-7.40 ± 4.36	0.002	-3.24 ± 1.95	-3.10 ± 2.21	0.24
**Motion53(X)**	1.51 ± 1.91	2.69 ± 1.49	0.013*	3.06 ± 1.52	3.84 ± 5.34	0.034	2.00 ± 1.75	2.15 ± 1.44	0.32
**Motion53(Y)**	-3.90 ± 3.04	-6.42 ± 2.95	0.02*	-5.55 ± 5.90	-15.56 ± 15.48	0.0001	-3.25 ± 3.31	-3.29 ± 3.70	
**Motion53(Z)**	-1.45 ± 2.52	-1.49 ± 0.80	0.45	-4.15 ± 5.56	-5.31 ± 5.54	0.01	-1.91 ± 3.54	-2.00 ± 3.90	0.57
**Motion75(X)**	-5.75 ± 2.99	-6.83 ± 1.50	0.03*	-8.79 ± 2.95	-11.60 ± 5.18	0.001	-4.47 ± 1.58	-3.87 ± 1.59	0.11
**Motion75(Y)**	12.36 ± 3.19	8.09 ± 7.67	< 0.001*	11.55 ± 5.33	7.34 ± 8.31	0.002	6.95 ± 2.79	7.10 ± 3.10	0.33
**Motion75(Z)**	8.96 ± 3.08	6.45 ± 1.94	< 0.01*	13.48 ± 5.49	10.47 ± 5.50	0.008	7.75 ± 2.85	8.12 ± 2.87	0.56

Motion31 (X), motion31 (Y) and motion31 (Z) respectively represent the motion changes of each joint on the X, Y and Z axes from the first posture to the third posture; Motion53 (X), motion53 (Y) and motion53 (Z) respectively represent the motion changes of each joint on the X, Y and Z axes from the third posture to the fifth posture; Motion75 (X), motion75 (Y) and motion75 (X) represent the motion changes of each joint on the X, Y and Z axes from the fifth posture to the seventh posture

^*^P < 0.05 showed statistical difference

### ROM of hindfoot from the third to the fifth posture (mid-stance phase)

Table 4 details the ROM of hindfoot from the third to the fifth posture. In the subtalar joint, the flatfoot had a higher ROM on X-axis (2.69 ± 1.49° vs 1.51 ± 1.91°, *p* < 0.05) and Y-axis (6.42 ± 2.95° vs 3.90 ± 3.04°, *p* < 0.05) than the non-flatfoot, but no difference was observed on Z-axis (1.49 ± 0.80° vs 1.45 ± 2.52°, *p* > 0.05, Fig. 7A). In the talonavicular joint, the flatfoot had a higher ROM on all the X-axis (3.84 ± 5.34° vs 3.06 ± 1.52°, *p* < 0.05), Y-axis (15.56 ± 15.48° vs 5.55 ± 5.90°, *p* < 0.05) and Z-axis (5.31 ± 5.54° vs 4.15 ± 5.56°, *p* < 0.05) than the non-flatfoot (Fig. 7B). However, in the calcaneocuboid joint, there was no difference between the non-flatfoot and flatfoot on X-axis (2.00 ± 1.75° vs 2.15 ± 1.44°, *p* > 0.05), Y-axis (3.25 ± 3.31° vs 3.29 ± 3.70°, *p* > 0.05) and Z-axis (1.91 ± 3.54° vs 2.00 ± 3.90°, *p* > 0.05) (Fig. 7C).

### ROM of hindfoot from the fifth to the seventh posture (MS to TO)

Table 4 also details the ROM of hindfoot from the fifth to the seventh posture. In the subtalar joint, the flatfoot had a higher ROM on all the X-axis (6.83 ± 1.50° vs 5.75 ± 2.99°, *p* < 0.05), Y-axis (8.09 ± 7.67° vs 12.36 ± 3.19°, *p* < 0.05) and Z-axis (6.45 ± 1.94° vs 8.96 ± 3.08°, *p* < 0.05) than the non-flatfoot (Fig. 7A). In the talonavicular joint, the flatfoot had a higher ROM on X-axis (11.60 ± 5.18° vs 8.79 ± 2.95°, *p* < 0.05), but a lower ROM on Y-axis (7.34 ± 8.31° vs 11.55 ± 5.33° *p* < 0.05) and Z-axis (10.47 ± 5.50° vs 13.48 ± 5.49°, *p* < 0.05), than the non-flatfoot (Fig. 7B). However, in the calcaneocuboid joint, there was no difference between the non-flatfoot and flatfoot on X-axis (4.47 ± 1.58° vs 3.87 ± 1.59°, *p* > 0.05), Y-axis (6.95 ± 2.79° vs 7.10 ± 3.10°, *p* > 0.05) and Z-axis (7.75 ± 2.85° vs 8.12 ± 2.87°, *p* > 0.05) (Fig. 7C).

## Discussion

The present study found that during the early- and mid-stance phase, excessive motion was observed in the subtalar and talonavicular joints in stage II AAFD; the motion of subtalar and talonavicular joints appeared to be in the dysfunction state during the late-stance phase; while the motion of calcaneocuboid joint showed no significant difference between non-flatfoot and stage II AAFD during the whole stance phase. According to a mass of studies focusing on single-plane fluoroscopy systems have examined the joint motion [22, 23]. Banks et al. [24] and Acker et al. [22] reported the accuracy of single-plane fluoroscopy system in measuring the movement of joint replacement prosthesis. The error of measurement based on the single-plane study in other planes perpendicular to this plane could be up to 0.65 to 4 mm, but the error of rotational measurement was small. However, the accuracy of single-plane 3D-to-2D registration technique was verified by RSA.

Among the 4 tarsals in the hindfoot, the talus had the largest rotation deviation, but its value was only 0.87 ± 0.32°, while the maximum rotation deviation in the subtalar joint was − 1.8 ± 2.33°. Therefore, the measurement of rotation in our study was very accurate, and the rotation deviation did not exceed the deviation results of the uniplanar X-ray measurement technique reported in the relevant literature [16, 22, 23].

Our findings revealed that, in the early-stance phase (HS to FF), the navicular and calcaneus relative to the talus was more dorsiflexed, externally-rotated and everted in the AAFD group than in the non-flatfoot group. It was mainly due to the fact that at the early stage of stance phase, the calcaneus was the first to touch on the ground, resulting in the calcaneus to eversion relative to the talus. With the continued weight-bearing, the talus shifted medially due to stress, so the calcaneus was also relatively externally rotated. Due to the laxity of the medial structures in AAFD patients (mainly the laxity of the spring and deltoid ligaments), excessive plantarflexion and adduction occurred in the talus. With the advancement of gait to the mid-stance phase, the heel began to flatten and the foot tended to bear an increasingly higher weight. For the AAFD, as the load increased significantly, the arch continued to collapse, causing the talus continued to move medially, and the scaphoid and calcaneus relative to the talus dorsiflexion and external rotation, resulting in the flatfoot navicular to have greater dorsiflexion and everted and externally rotated than the normal navicular. Nevertheless, due to the small rotation amplitude of the calcaneus itself, no significant difference was observed in the rotation of calcaneus of the AAFD group during this period. After the mid-stance phase, the foot would begin to enter the late-stance phase (off-ground stage). The degree of internal rotation and inversion of the navicular in non-flatfoot group was greater than that of the AAFD group due to the normal function of the posterior tibial tendon and the navicular. Similarly, the degree of internal rotation and inversion of the calcaneus was also greater than that of the AAFD group. Conversely, due to the insufficient medial support of flatfoot, the degree of plantar flexion of the calcaneus and navicular was greater than that of the non-flatfoot in the off-ground process. However, no significant difference in the movement of flatfoot and normal calcaneocuboid joints throughout the stance phase was found, which also confirms that the instability of the hindfoot of stage II flatfoot is mainly attributed to the subtalar and talonavicular joints, while the lesion has not yet involved the lateral calcaneocuboid joint. By aiming at stage II AAFD and non-flatfoot groups as the study subjects and the stance phase of gait as the main process, our study confirmed that there was hyperactivity in the subtalar joint and talonavicular joint of stage II AAFD group compared with non-flatfoot group in the early- and mid-stance phase, and the motion of subtalar joint and talonavicular joint in stage II AAFD group showed a state of decompensation in the late-stance phase. There was no significant difference in stage II flatfoot and normal calcaneocuboid joint motion throughout the phase. This is consistent with the conclusions regarding the subtalar and talonavicular joint instability derived from quasi-static studies of stage II AAFD [17].

Limitation of the present study should also be acknowledged. Firstly, stage II AAFD was not further subdivided into phase IIa but generally classified as one category for analysis. Second, the participants can only walk with this limited speed (0.5 m/s) which is less than the half of the typical speed due to experimental condition limitation, Therefore, the heel impact is partially missed which might influence on the accuracy of the results. Last but not least, the experimental sample size was relatively small, which might bias the real results. However, we believe that the current research improves our understanding of the kinematic etiology of AAFD. Future direction will be more advanced instruments that can better detect gait posture during walking, while increasing the sample size and improving the accuracy of the measurement.

## Conclusions

During the early- and mid-stance phase, excessive motion was observed in the subtalar and talonavicular joints in stage II AAFD. During the late-stance phase, the motion of subtalar and talonavicular joints appeared to be in the dysfunction state. The current study helps better understanding the biomechanics of the hindfoot during non-flatfoot and AAFD condition which is critical to the intervention to the flatfoot using conservative treatment such as insole or surgical treatment for joint hypermotion.

## Data Availability

Please contact author for data requests.

## Abbreviations

AAFD: Adult acquired flatfoot deformity
TNJ: Talo-navicular joint
STJ: Subtalar joint
CCJ: Calcaneo-cuboid joint
HS: Heel-strike
FF: Foot-flat
MS: Midstance
HO: Heel-off
TO: Toe-off
IRB: Institutional Review Board
RSA: Radiographic stereophotogrammetry analysis
ROM: Range of motion
